# Evaluation of Castor Oil Cake Starch and Recovered Glycerol and Development of “Green” Composites Based on Those with Plant Fibers

**DOI:** 10.3390/ma9020076

**Published:** 2016-01-27

**Authors:** José Luis Guimarães, Ana Cristina Trindade Cursino, Cyro Ketzer Saul, Maria Rita Sierrakowski, Luiz Pereira Ramos, Kestur Gundappa Satyanarayana

**Affiliations:** 1 Setor de Educação Profissional e Tecnológica; Universidade Federal do Paraná—UFPR, Centro Politécnico, Rua Alcides Vieira Arcoverde, 1225, Jardim das Américas, Curitiba 81520-260, Brazil; jluisguimaraes@gmail.com; 2 Departamento de Química, Universidade Federal do Paraná—UFPR, Centro Politécnico, P. B. No.19081, Jardim das Américas, Curitiba 81531-980, Brazil; anatcursino@gmail.com (A.C.T.C.); mariarita.sierakowski@gmail.com (M.R.S.); luiz.ramos@ufpr.br (L.P.R.); 3 Departamento de Física, Universidade Federal do Paraná—UFPR, Centro Politécnico, P. B. No.19044, Jardim das Américas, Curitiba 81531-980, Brazil; cyro.saul@gmail.com

**Keywords:** fibers, castor oil cake, starch, glycerol, thermo-molding, green composites

## Abstract

Continuous efforts are being made in some countries for the recovery of crude glycerin (RG/CG) and castor oil cake (COC), the two byproducts of biodiesel production. These are expected to help, not only in addressing environmental safety, but also in adding value to those byproducts, which otherwise may go to waste. Finding ways to utilize those byproducts underlines the main objective of this study. This paper presents the evaluation of (i) COC, glycerin and banana and sugarcane fibers for moisture content; (ii) COC for structural and thermal properties; and (iii) CG for its chemical characteristics. The possibility of using COC and CG with the selected fibers as reinforcement in the development of bio-composites is attempted through thermo-molding. Results revealed enhanced mechanical properties for these composites. The obtained results are discussed in terms of the observed morphology.

## 1. Introduction

Recognizing the depletion of petro-chemical resources and growing environmental awareness, researchers dealing with composites have been searching for the replacement of expensive and non-renewable petroleum based synthetic fibers and polymers. Beside these reasons, other petroleum based materials’ demerits have to be taken into account such as low biodegradability and energy intensive manufacturing. Contrary to the above, natural polymers and lignocellulosic fibers from different bio-renewable resources possess unique intrinsic properties such as abundant availability, biodegradability, environmental friendliness, flexibility, ease of processing and comparable mechanical properties. Thus, this new kind of composite is called “biodegradable”, “green” or “eco-friendly materials”.

These materials can be recycled or be triggered to biodegrade, if required. Thus, these new natural materials are becoming indispensable components in the development of low-cost eco-friendly composites exhibiting acceptable specific strength, low density, high toughness and good thermal properties.

This development seems to be one of the most rapidly emerging fields of research in polymer science and engineering. This is evident from the increasing publications of book chapters, review papers and original research papers all over the world, highlighting the advantages of using natural polymers and lignocellulosic fibers. Due to the limitation of space, only a few of these are mentioned here in a chronological order [1,2,3,4,5,6,7,8,9,10,11,12,13,14,15,16,17,18,19,20,21,22,23,24,25,26,27,28,29,30,31,32,33,34,35,36].

Following this trend, this study has focused on the evaluation of castor oil cake (COC) and recovered glycerin (RG) as potential raw materials in the preparation of “green” composites. While the former has been obtained as a residue from castor oil manufacture, the latter has been obtained as a byproduct during biodiesel production. It is called “crude or recovered glycerin” (hereafter designated as RG throughout the paper unless otherwise stated), since it is a mixture of 81%–85% glycerol with the remaining consisting of water, waste oil/fat and soaps. These are all alkaline trans-esterification reaction byproducts [37,38].

The reasons for this study on the evaluation of castor oil cake (COC) and recovered glycerin (RG) are as follows:

(i) With advanced biofuels representing at least 2.5% of energy consumption in transport by 2020, the demand for biofuels has triggered greater demand for biodiesel all over the Europe [39] and also in Brazil. For example: the biofuels production in Brazil has increased tremendously from 2006 (about 69 million liters) to date (27.6 billion liters during 2015–2016) [40] with possibility of the whole production to be consumed locally [41] Thus, with increasing global production of biodiesel, the amount of the RG would also increase with about 20 metric tons of oils being chemically processed annually [38].

**Figure 1:**
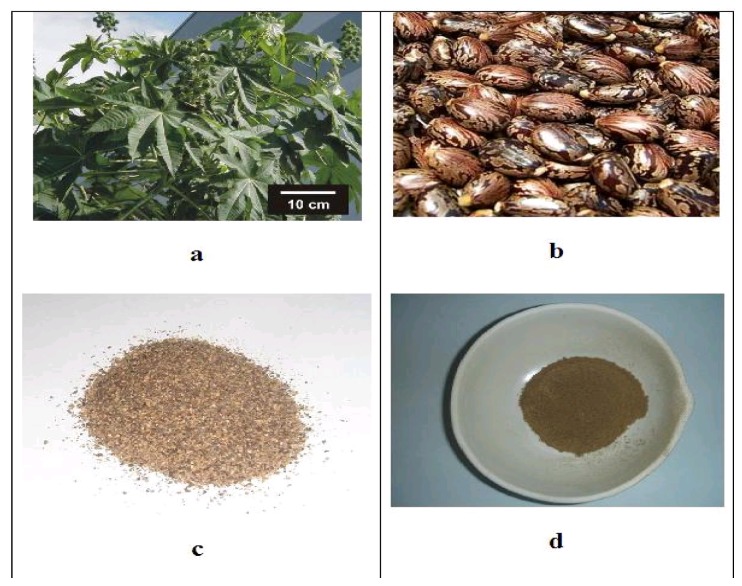
(**a**) Castor plant; (**b**) Castor beans; (**c**) Castor bean cake after extraction of oil; (**d**) Dry castor bean cake after grinding in a vibratory mill.

Besides, with very little room for extending the use of glycerin in conventional applications such as in pharmaceutical and cosmetics industries, a large amount of surplus RG will become available globally. This issue needs to be addressed considering that the amount of impurities present in RG does not makes it suitable for use in the main glycerol consuming market, such as pharmaceutical, cosmetics and aliment industries. This also creates a logistic problem since the lack of purification processes demands for storage and transportation centers to absorb the production. One solution relies on its use, without purification, as a plasticizer for starches in substitution of commercial glycerol. The reason for this lies in stability and compatibility provided by glycerol with packaging chains of hydrophilic starches [5]. Already, such a study has been successfully carried out by the authors in the development of a corn starch based green composite [27].

(ii) The castor oil cake (COC) is a solid residue obtained after oil extraction from the seeds of *Ricinus communis* L (Figure 1a,b). This seed contains, on average, 25–55 wt % oil [42,43]. Figure 1c,d shows the cake after oil extraction and in the dry form after grinding in a vibratory mill, respectively.

Further, this cake is reported to be rich in protein (21%–48%), which can only be used as soil fertilizer [44]. However, there has been an increase in a variety of other cultures having low cost for detoxification and production as in the case of cotton, soybean, *etc*. Hence, there is need to find other ways to use this cake, since it is reported to have about 28%–49% plasticizable components (high protein) and 26%–29% strengthening material (crude fiber) [42,43].

It also suggests its potential as a protein source to produce biodegradable materials. The ease of cross linking due to the amino acid richness and the good film properties associated with the protein content [45] make the COC an attractive matrix for developing “green” composites with suitable reinforcements. In fact, many biodegradable materials have been developed with similar protein fractions [3].

(iii) There are also reports regarding the use of COC as raw material in the preparation of biodegradable materials. Being a protein source, and also as a filler, it is reported to be of useful raw material for the preparation of composite materials with improved properties [26,42,43,46,47,48,49,50,51,52,53,54,55,56]. Some details of these studies are briefly mentioned in the following: a patent was filed regarding the composition and process to obtain composite materials containing conventional thermoplastics, a biodegradable biopolymer (Polyhydroxyalkanoate), with COC as filler [26]. The proposition of producing thermoplastics is corroborated by the highest reported Young’s modulus (YM) value for cassava starch, containing fibers and sugars (both being capable of strengthening the matrix); other similar materials being industrial starch and that derived from its roots. It was observed that the YM decreased with the increasing amount of plasticizer (glycerol) [15].

Recently, the preparation of extruded biocomposites containing castor oil cake as filler in poly hydroxyl butyrate (PHB) blended with low density polyethylene (LDPE) and their characterization by mechanical properties and morphology was reported by Burlein and Rocha [51]. It was observed that the Young’s modulus of LDPE increased with the addition of PHB or COC, while the other tensile properties and impact resistance were deteriorated. The observed low properties were explained as due to the poor interfacial adhesion between the constituents. Another study has reported preparation of films of both the matrix and its composites [48]. The matrix was based on the proteins extracted from castor oil cake through cross linking it with glyoxal, while their composites reinforced with 5–12.5 (per 100 g of protein with and without 5–30 g glyoxal)cellulose pulp fibers extracted from eucalyptus for their possible use in agriculture. All the physical and mechanical properties studied showed improvement with the incorporation of fibers, which have been explained based on the tested composite morphology.

(iv) A research group at the Federal University of Parana (UFPR)-Curitiba-PR-Brazil, in association with other agencies, has taken up several projects to expand uses for the COC in particular. The overall objective of this research group is to formulate some attractive propositions through composite technology to explore new utilization routes for co-products of the biodiesel industry with or without other agro industrial byproducts. Hence, this concept is important for countries like Brazil, where its Northeastern region produces large quantities of castor seeds and uses the castor oil in several processes including the production of biodiesel. Such efforts already reported include the use of COC (a) either fully or partly substituting conventional starches to develop biodegradable composites using lignocellulosic fibers such as banana, sugarcane bagasse *etc.* [43,49,53,54,55]; and (b) mainly as matrix after converting it into a thermoplastic for reinforcing lignocellulosic micro fibers, or wood derived nano cellulose [56].

(v) Finally, there are published reports dealing with the physical, mechanical and thermal properties of various lignocellulosic fibers of Brazil [52,57].

Considering all the above mentioned facts with a few published reports and some patents regarding the use of COC, very little attention is being given in Brazil to the use of the agro-industrial byproducts, such as COC, crude glycerin and lignocellulosic fibers. Therefore, this paper presents (a) the characterization of COC and RG with regard to some of their physical and thermal properties; and (b) evaluation of their “green” composites prepared by thermo molding with the incorporation of banana and sugarcane bagasse fibers.

## 2. Experimental Section

### 2.1. Materials

The COC supplied by the Azevedo Oils Industrial and Commerce Ltd. (Itupeva, SP, Brazil) was used in the present work. This COC was reported to have a pH of 6.5 when it was mixed with water in 1:10 proportion. It consisted of 75% organic matter, 9% moisture content, 5% nitrogen and about 14% inorganic residues [58]. This cake along with glycerol as plasticizer was used as matrix in view of its high content of protein and organic matter [42,43].

Recovered glycerol (RG) was used as plasticizer. This was obtained as a byproduct of biodiesel pilot plant [Technological Institute of Paraná (TECPAR) Curitiba, PR, Brazil] by ethanolysis of soybean oil using sodium hydroxide as the reaction catalyst precursor was used [27]. Reported composition of this RG was 81.7% glycerol, 4.4% water, 0.03% of ethanol and traces of methanol, as well as the remaining as soaps and the alkaline catalyst [59].Some experiments were also carried out with pure glycerol (PA).

With the COC + RG matrix fibers of both banana and sugar cane bagasse were used as reinforcements in the present work. The latter fibers are also called “bagasse fibers” in this paper. These fibers were procured followed by their conditioning as reported earlier [52]. In brief, banana sheaths were obtained from small logs cut from a banana plant. Then, these sheaths were scrapped manually with a knife to obtain the fibers followed by drying for five days in an open environment. On the other hand, the bagasse fibers used were from a local ethanol manufacturing industry.

### 2.2. Methods

#### 2.2.1. Preparation of Castor Oil Cake as a Matrix Material

##### Extract of Excess oil from COC (Treatment to COC)

Due to the limited reflux processing capability, the COC sample was divided into three fractions (I, II & III).During the fabrication of laminates using the COC samples, surplus oil was observed on the molds due to heating and pressing. In fact, this excess oil which is probably linked to the reduced efficiency of the oil industry extraction process, worked as demoulding agent. Even though this excess oil apparently did not affect the composite fabrication process, it was decided to remove it using a Soxhlet apparatus and hexane as the extraction solvent, aiming to improve the composite samples’ appearance. The resulted COC was another type of COC and this process is hereafter referred to as “treatment” to COC. Then, the COC was washed and dried to remove the residual oil using an organic solvent. This COC is hereafter referred to as COC (MT) to designate milled and treated samples.

##### Castor Oil Cake for Matrix

In the first place, the obtained COC was kept in a hot air oven maintained at about 70 °C for drying at temperatures of 65–70 °C. A vibratory mill was used to grind this dried COC followed by ball milling it to obtain particulates of <40 mesh in size. This ground COC was dried for 24 h at 85 °C in hot air oven. Through sieving process, the ground and dried COC was separated into particles of >80 mesh, 60–80 mesh and <40 mesh size. It was observed that about 6.8% of the sieved powder greater than mesh size of 60, about 11.2% was in the range of mesh size of 40–60 and the balance 82% was less than 40 mesh size. The last powder portion (<40 mesh), hereafter termed as “MST” (milled and untreated) was used in the preparation of composites in view of its greater percentage. In the second set of samples, treated COC (MT) was used to prepare the laminates. The product was oven dried at 65–70 °C for 72 h. These samples were called here after as “MT” (milled and treated), which was also used in the preparation of some composites to understand the effect of treatment of COC.

#### 2.2.2. Characterization of Castor Oil Cake and Glycerol/Recovered Glycerol (RG)

Studies of COC with and without treatment (MT and MST) were carried out by X-ray diffraction (XRD) method using a Shimadzu (Kyoto, Japan) diffractometer model XRD 7000 with CuKα radiation (λ = 0.15418 nm) with operating conditions of 40 kV and 20 mA.

The thermal analysis of raw materials provides important information about the moisture content and maximum temperature for processing these matrices. Accordingly, thermal stability and water loss after heating of COC were determined using Metler-Toledo TGASDTA-851E equipment (Columbus, OH, USA). The samples were subjected to heating at 800 °C, with 10 °C·min^−1^ as the heating rate, under a dynamic flux of air (50 mL/min).

Moisture content of lignocellulosic materials is an important property to be understood, since moisture plays a decisive role during the composite processing and also influences their tensile properties. Hence, prior to preparing the laminates of both the matrices and composites, their moisture content was measured following standard method.

FTIR studies of two types of glycerol were carried out by mixing 10 mg of each sample with 1 mg of KBr pellets with a view to reduce the peak intensity. An Excalibur Bio-Rad FTIR spectrophotometer (Hercules, New York, NY, USA), Model FTS 3500 GX was used for this purpose using a resolution of 2 cm^−1^ and accumulation of 32 scans.

#### 2.2.3. Preparation of Lignocellulosic Fibers for Use as Reinforcements

The best fiber parameters for composite preparation were found to be 67–300 µm (average diameter) and an average length of 1.5 mm for banana fiber. Values of these for bagasse fibers were 200–320 µm and 1.5–3.0 mm, respectively. Since the two types of fibers had different lengths, they were milled in a vibratory ball mill for 1–3 h, which resulted in fibers of uniform length (1.5–3 mm). The reason for this was to compare the properties of prepared composites using identical types and sizes of natural fibers, similar to the ones used earlier by the authors in the development of corn starch based composites [27]. These fibers were then dried in a hot air oven for 72 h at 65–70 °C.

Earlier studies reported lower tensile properties with the use of these fibers without any surface treatments in the preparation of COC + RG or starches (corn and cassava) + RG based composites compared to those of respective matrices [54]. Hence, in this study, both the fibers (banana fibers and sugarcane bagasse fibers) were given surface treatments. Both these fibers were treated by refluxing in n-hexane to remove the wax and hydrophobic components. These fibers were designated as “BT” and “SBT”, respectively. Both of these surface modified fibers were dried for 24 h at 65 °C in a hot air oven before using them in composite preparation.

#### 2.2.4. Fabrication of Matrix Laminates and Bio-Composites

Details of processing of COC based composites studied in this investigation are patented [53] and, therefore, are only briefly given here: Laminates of matrix (COC + RG) and its composites with banana and bagasse fibers (both treated) were prepared under the temperature and pressure controlled conditions as follows: the conditions used were based on authors’ earlier experience in the processing matrix of COC + pure glycerol (PA) or corn starch with 30% PA or 30% RG [27,54,55]. These conditions had revealed ease of processing and sample homogeneity with the amount of plasticizer used as ideal for processing. Accordingly, the same amount of RG (30%) was used in this study also. For this purpose, two types of mixers, viz., a ball mill and a mechanical mixer with a steel vat and blades were used to mix COC and RG. For preparing composites of COC + RG matrix, sieved fractions of 25% banana fiber and 20% of bagasse fibers were added to the matrix followed by homogenous mixing using a mechanical mixer having a metallic container. Selection of different fiber content (25% BT and 20% SBT fibers) in this study was based on the following: (i) earlier reports of better strength properties for composites of corn starch or COC + RG containing surface modified bagasse fibers (SBT) than those containing surface modified banana fibers (BT) [27,54]; and (ii) comparison of properties of the composites prepared in this study with those of COC composites containing treated fibers of both banana and sugarcane bagasse, but prepared by controlled conditions as reported earlier [54,55].

The mixture was then homogenized in a 316 L stainless steel mould with dimensions of 220 × 220 mm, and following thermo-molding process with a hydraulic press (SOLAB make) having controls for heating and pressing. The load range used was 4–9 tons (pressure range of 200–350 kgf/cm^2^). The pressing was done in a temperature range between 140 and 190 °C. The temperature in the press was measured using two thermocouples with one in each plate of the mold. Throughout the experiment, the temperature was maintained within ±3 °C of the specified molding temperature. The mixture in the mold was slowly heated to 150–160 °C with a heating rate of 10 °C/min and kept at this temperature and load for 3 min. This was then followed by cooling the sample till its temperature reached about 60 °C, where the sample stayed for about 12 h. Finally, the sample was cooled to room temperature, which resulted in laminates of 120 mm × 120 mm × 25 mm in size were used for further evaluations.

A few laminates of COC + 30% pure glycerol (PA) were also prepared for comparison of fractographic studies with those of COC + 30% RG.

Table 1 lists designation (explained at the footnote of the table itself) of these samples indicating composition and combinations of matrix materials, plasticizer and the fibers.

**Table 1:** Details of composite samples prepared.

Sample	Constituents of Matrix (%)	Reinforcing Fiber (%)
COC70 G30	70 Castor oil Cake-30 Pure Glycerol	0
COC70 RG30	70 Castor oil Cake-30 Recovered glycerol (RG)	0
COC45RG25BT	45 Castor oil Cake-30 Recovered glycerol (RG)	25 Banana Treated (BT)
COC50RG20SBT	50 Castor oil Cake-30 Recovered glycerol (RG)	20 Bagasse Treated (SBT)

COC: Castor oil cake; G: Pure/Commercial Glycerol; RG: Recovered glycerol; BT: Treated banana fiber; SBT: Treated sugarcane bagasse.

#### 2.2.5. Characterization of Prepared Composites

Various laminates prepared as mentioned above were kept in desiccators maintained at about 50% relative humidity (RH) with a view to equilibrate them in this condition. Then, they were subjected to further evaluations. In general, the COC + RG composite samples were found to be more homogeneous. This suggested that the amount of plasticizer used was ideal for processing as well as from a morphology perspective, as it will be seen later.

Following the ASTM standard [60], tensile samples were cut into required shape and dimensions using CO_2_ laser cutter [GRAVOGRAPH LS-100 (30 W)] using 0.5 mm·min^−1^ cutting speed.

Following the ASTM standard [60], tensile tests on prepared matrix as well as composite samples were carried out using two testing machines (EMIC (DL-2000) and INSTRON (Model-5960)), with a 5 kN load cell and test speed of 1 mm/min, This was done with a view to compare the values using different testing machines.

Values of all the properties of raw materials studied were obtained by averaging values of 3 samples tested. However, in the case of tensile testing, except for the matrix (COC + 30RG), 5 samples were used for each of the materials studied. This is because very few samples could be obtained for tensile testing the matrix COC + 30 RG, as many samples of this matrix broke when attempting to cut them using the laser cutter as they were found to be too fragile, similarly to a ceramic material.

Fractographic studies of tensile tested matrix laminates of COC+ RG and their composites with banana and sugar cane bagasse fibers were carried out using a JEOL (Peabody, MA, USA) scanning electron microscope (SEM) at 15 keV after gold coating to avoid sample charging.

## 3. Results and Discussion

### 3.1. Extraction of Excess Oil from Castor Oil Seed

About 2.5% oil yield was found in all three fractions, while it has been reported that only about 1.5% of residual oil was good quality castor bean following double extraction. In fact, Azevedo Oil Company specifies the castor bean oil residue at 1.5% only [58]. This was favored for fast assimilation by the soil [44]. The observed difference may be due to in-homogeneity in samples and may be very important, because the company commercializes millions of liters of oil per year.

### 3.2. Moisture Content

The moisture contents of the raw materials (COC, RG and two plant fibers) were found to be 10.8%, 9.3%, 8.6%, and 9.2%, respectively, for COC, RG, banana fibers and bagasse fibers. The values obtained are within the range that is normally observed for any lignocellulosic material (~11%) at room temperature (65% RH). Also, the values of two fibers were similar to those reported by the authors elsewhere [52].

### 3.3. Thermal Analysis

Initial trials showed that the moisture content of matrix (>9%) was of fundamental importance during processing of bio-composite samples. This is because, the excess water turned into steam, leading to surface bubble formation, which increased the brittleness of laminates. Therefore, this is vital information to control and to define the processing temperatures, which should not exceed 200 °C in this case.

**Figure 2:**
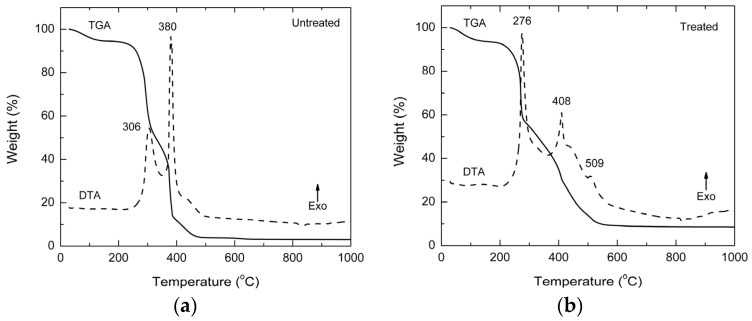
TGA/DTA curves of castor oil cake samples: (**a**) without treatment (MST); and (**b**) with solvent extraction treatment (MT).

Figure 2 shows TGA/DTA curves for the COC samples, both untreated (MST) and treated (MT).

The curves show that both COC samples are different*.* For example, the untreated COC (MST) (Figure 2a) showed a mass loss of 5.5% at 170 °C due to the evaporation of physisorbed water. Another mass loss of 46.7%, associated with an exothermic peak centered at 306 °C, was observed between 170 °C and 340 °C. A further mass loss of about 44.7% was observed between 340 °C and 1000 °C and this is associated with the exothermic peak centered at 380 °C. These intense peaks masked the peak of water loss within the background. If the DTA curve expanded in its y axis, a small endothermic broad peak would appear around 104 °C. The remaining 3.1% of residues (ash) may be associated to soot formation, as well as to inorganic residues such as phosphates, sulfates and silicates. The presence of inorganic residues is understandable, since the castor bean plant is a very demanding in terms of soil quality. This in turn underlines the fact that nitrogen, phosphorous and potassium, as well as a pH of 6–7 are essential to ensure productivity. The amounts of nitrogen, organic matter and humidity in the present samples are similar to the amounts reported by Azevedo Industrial and Commerce Oil Ltd., Sao Paulo, Brazil, and are in accordance with that expected for Brazilian plants.

On the other hand, the treated COC (MT) (Figure 2b) showed a 6.5% loss of mass at 170 °C due to physisorbed water. Another mass loss of 45.6% is observed between 170 °C and 340 °C and it is associated with an exothermic peak centered at 276 °C. Further mass loss of about 43.6%, observed between 340 °C and 1000 °C, is associated with an exothermic peak centered at 408 °C, as well the shoulders (small peaks) at 509 °C. The remaining 4.3% ash content is normally expected for lignocellulosic materials. It is worth to be noted that the treated COC sample showed a higher content of ashes, indicating that the solvent treatment was efficient to remove organic matter (oil) from the sample. The ash content difference mentioned above between the MST and MT COC samples cannot be considered as significant, since it varies according to the place from where the sample was taken. It would be higher if the samples were obtained from older plants or planted on shale, but always it would be about 3%–4%. The ashes just indicated the sample mineral content. The complex decomposition profile, which has the first main combustion peak higher than the second one (inverted in the non-treated sample), suggests that the solvent treatment also interfered in the sample structure. This effect could be interesting for two reasons. The first one is the extraction of oil and the second is that more homogeneous phases were generated. The latter resulted in a structure which was more suitable to interact with the plasticizer. It should be noted that a more homogeneous structure would be obtained when the entire solid is plasticized with glycerol. This homogeneity could be observed when the samples did not show phase segregation as observed in the morphology studies (See Section 3.6). This leads to improved mechanical properties as will be discussed later.

DTA/TG studies on banana, and bagasse fibers are already reported by the authors [52]. The mineral ashes found in these fibers were 2.9%, 2.9% and 0.8%, respectively.

### 3.4. FTIR Spectroscopy Studies of Recovered Glycerol (RG)

**Figure 3:**
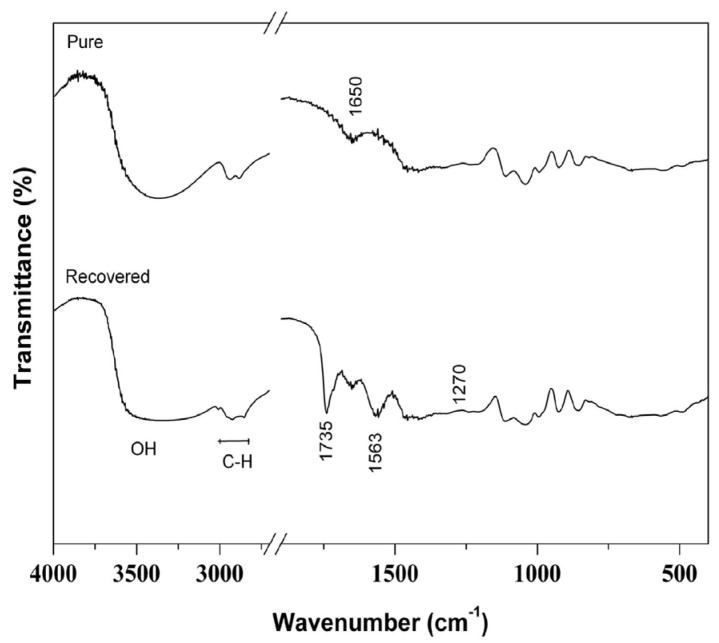
FTIR spectra of pure glycerol and recovered glycerol (RG).

RG from biodiesel production has no value as fuel except for the impurities from trans-esterification reactions such as ester, fatty acids, soaps and water [61,62]. FTIR spectra were recorded in order to verify impurities in the RG by comparing with analytical grade glycerol. The spectra in Figure 3 of RG and pure glycerol have a wide band between 600 m^−1^ and 3000 cm^−1^ caused by stretching of O–H bonds with either inter or intra-molecular hydrogen bonds, that could also be formed by water molecules, whose angular deformation was seen at 1651 cm^−1^. In fact, water was observed in all samples.

In fact, water was observed in all samples. Another intense band that appeared only in the RG was the one at 1563 cm^−1^, very common for carboxylate that accumulated as residues from alkaline trans-esterification or for the presence of ester products from incomplete separation from glycerin.

Though small (but, can be seen clearly if the scale is expanded), an additional O–H vibrational mode for the RG was also seen at 1270 cm^−1^ (See Figure 3). This was from OH groups in glycerin and probably from residual fatty acids, whose intense C=O stretching appeared at 1735 cm^−1^. The long alkyl chain of fatty acids is reflected in the 2925 and 2852 cm^−1^ region corresponding to axial deformation of C–H bonds in secondary (–CH_2_–) and primary (–CH_3_) carbon atoms. These are common to many organic substances.

### 3.5. X-ray Diffraction Studies

**Figure 4:**
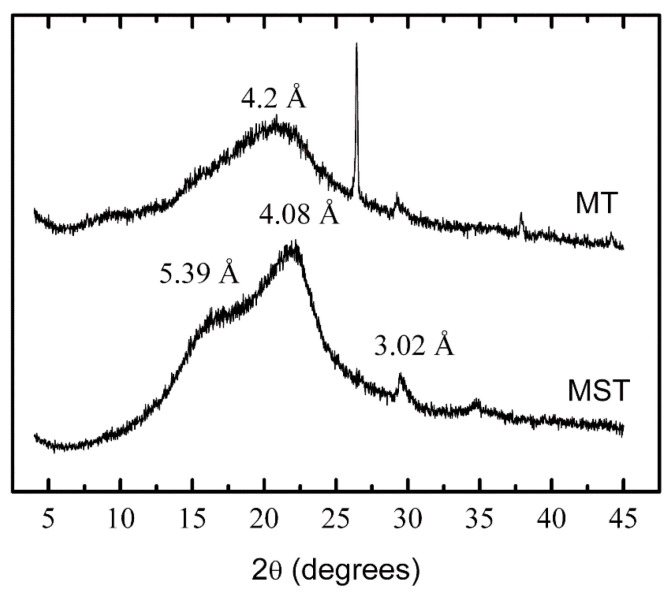
X-ray diffraction patterns of untreated (MST) and treated (MT) castor oil cake.

Figure 4 shows X-ray powder diffraction patterns of both untreated (MST) and treated (MT) COC.

It could be seen that the diffraction patterns of the samples without treatment (MST) showed the broad diffractions peaks (low crystallinity), at 5.39 Å, 4.08 Å and 3.02 Å. This could be attributed to a superposition of native starch and cellulose type I structure, typical of native cellulose of superior plants. The first peak at 2θ = 16.5° was followed by a strong peak at 2θ = 21° (high crystallinity). The high crystallinity peak may be due to intra-molecular interaction ordering between amino proteins and/or polysaccharide such as cellulose. The low crystallinity peak may be mostly related to cross linking [27]. The third broad peak of low intensity at 2θ = 40°–50° also indicate intra-molecular interactions involved in sample crystallinity.

On the other hand, after the treatment, the X-ray diffraction pattern of COC (MT) was dominated by a broad amorphous halo, showing the peak at 2θ = 15° and then a smaller peak at 2θ = 40°–50° and the third broad peak at 2θ = 21°. This was almost similar to those observed for the sample before treatment (MST). However, there were some differences in the peak sizes. While peak heights decreased, they were also broadened due to the treatment by the organic solvent. This suggests possibility of the removal of oily residues and other carbon chain materials reducing the crystallinity due to possible influence of inter-chain interactions. The sharp diffraction peaks in both samples were due to the aluminum sample holder. The observed sharp peak at 2θ = 26° only in the treated castor bean cake (MT) could be attributed to an inorganic salt impurity (5%), which might arise due to treatment methods of the cake.

### 3.6. Tensile Properties

**Figure 5:**
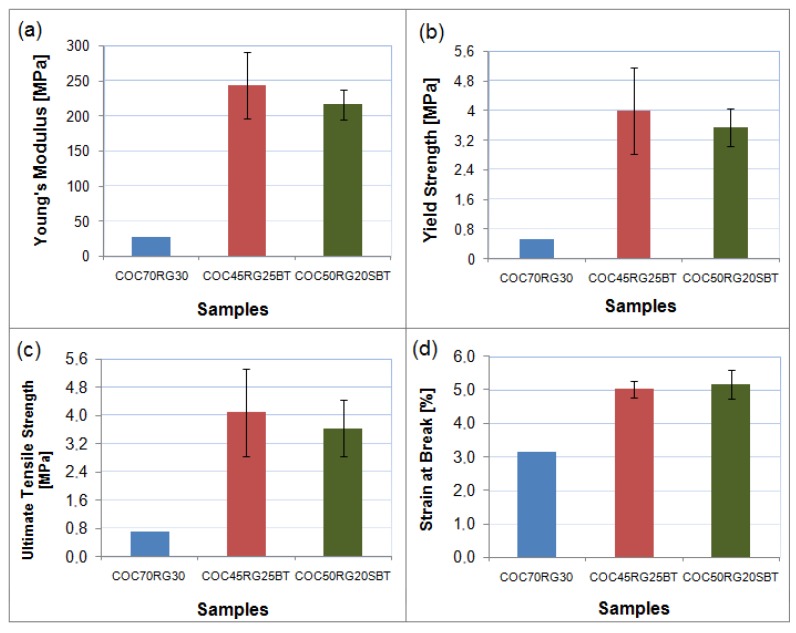
Plots of tensile properties of COC + RG with banana fibers and sugarcane bagasse fibers.

Figure 5 shows the plots of tensile properties (Young’s modulus (YM), Yield strength (YS), ultimate tensile strength (UTS) and strain at break) of all the samples prepared in this study. It may be noted that no standard deviations are shown in the case of the matrix consisting of COC + 30 RG since only two samples could be tested due to the reasons given earlier (Section 2.2.5).

From the figure, it can be seen that fiber incorporation has significantly improved all the tensile properties of the matrix irrespective of the fiber type. This suggests a good chemical and structural compatibility between the matrix and the fiber (cellulose chains) indicating very good adhesion between the two constituents. This enhancement of desired properties is independent of type and nature of matrix.

This observation is different from that observed for starch including corn starch + RG composites containing similar plant fibers [1,6,9,10,24]. In those cases, incorporation of plant fibers including the fibers used in this study into the plasticized starch improved only the Young’s modulus and yield strength, but without any changes in the tensile strength, and a decrease in the percentage of elongation at break over that of the matrix (starch + glycerol) was observed. In fact, the improvement in Young’s modulus in these systems was also attributed to deplastification of starch caused by the partition of glycerol between the composite constituents (matrix and fibers) [19].

Further, one can also see that values of YM, UTS and YS of 25% banana fiber composites with COC + RG were marginally higher than those of 20% bagasse composites with the same matrix. However, values of strain at break were very similar. From this, one may expect that bagasse fiber composites may be better in terms of tensile properties when an equal amount of both fibers are used.

### 3.7. Fractographic Studies of Castor Bean Cake and Its Composites

**Figure 6:**
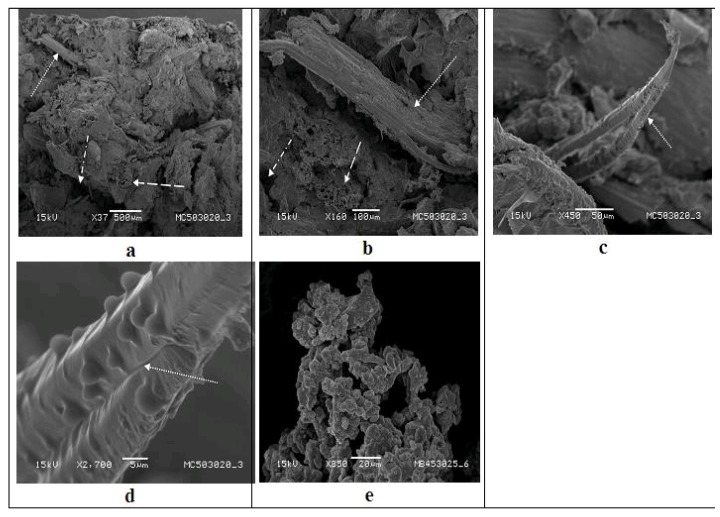
SEM photographs of castor oil cake—recovered glycerol (RG)—sugarcane bagasse fiber after the tensile test: (**a**) panoramic view of the fractured surface showing rough surface, fibers, cracks and voids; (**b**) region showing one fiber lying parallel and other embedded fractured fiber in the matrix; (**c**,**d**) pulled-out fiber covered with plasticizer (at two magnifications); (**e**) non-structured starch showing small polymorph granules.

**Figure 7:**
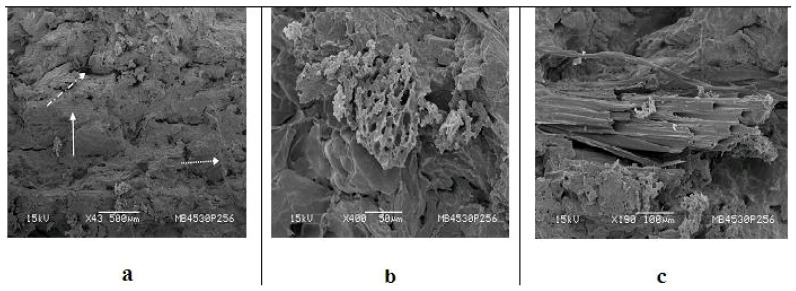
SEM photographs of COC—pure glycerol-banana fiber composite after the tensile test: (**a**) panoramic view of surface showing matrix, fibers and some cracks; (**b**) zoomed image of the encircled region near the crack edge seen (**a**); (**c**) fractured fiber embedded in the matrix.

**Figure 8:**
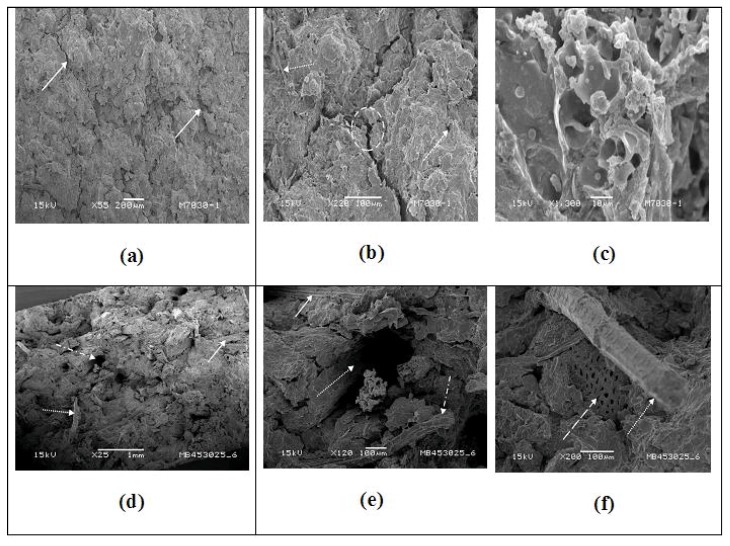
SEM photographs of 70 castor oil cake—30 recovered glycerol (RG) and its composite with banana fiber after the tensile test. (**a**) rough surface with cracks; (**b**) zoomed image of the region indicating the cracks shown in (**a**); (**c**) higher magnification of zoomed region of encircled region in figure (**b**); (**d**) panoramic view of the fractured surface of the composite showing rough surface, fibers and voids; (**e**) higher magnification of a region of (**a**) showing more cracks in the matrix, embedded failed fiber; (**f**) high magnification image showing pulled-out fiber with plasticizer coating and non-structured starch showing small polymorph granules.

Morphologies of composites of COC + 30% pure glycerol (PA), COC + 30% RG containing banana fiber and sugarcane bagasse fibers after the tensile testing as observed in a scanning electron microscope are shown in Figure 6, Figure 7 and Figure 8. A point to be noted in these micrographs is that the emphasis is on clarity of the morphological features and, thus, not all of them are of the same magnification.

One general observation of fractured surfaces of composites containing different fibers is that their surfaces show a dark brown color tending to dark. This suggests the effect of fibers on the matrix morphology. As evident from the figures, it becomes evident that the type of fiber dictates the morphology of the composite rather than that of matrix. This observation is similar to those made in earlier reports [7]. A heterogeneous structure and some small pores as seen in these figures suggest the presence of a second phase having a somewhat globular structure. An understanding of the observed fractographs of composites through the (i) effect of type of glycerin; and (ii) influence of fibers used with each type of glycerin is given below. It may also be noted that the photographs were taken after focusing on the central feature of the image at very high magnification. There are some regions out of focus, which is due to the topography of the samples exceeding by far the SEM depth of focus.

#### 3.7.1. Effect of Type of Fibers Used

In the case of bagasse fiber composite with COC and RG, a different type of fracture surface was observed as evident from Figure 6. A panoramic view of a fracture surface shown in Figure 6a reveals a very rough surface compared to that with banana fiber, mostly due to the shorter length of bagasse fibers with some pull outs (shown by dotted arrow). These fibers appear to become more homogeneously mixed and uniformly distributed than banana fibers. Some areas (broken line arrow) show structure similar to folded tissues along with fiber pull out holes (arrow with broken lines with dots). One can also see micro voids, fiber pull out holes (arrow with broken lines with dots) and fractured fiber surface (broken arrow) perpendicular to the composite fracture surface matrix, as well as fiber splitting (dotted arrow) (Figure 6b). Such fiber splitting and fractures have been observed earlier in composites fabricated with 6 MPa molding pressure [8].

Complete coating of the fiber surface by the matrix (arrow) can be seen at higher magnifications in Figure 6c,d. This indicates good interface between the matrix and the fiber as observed earlier in corn starch containing bagasse fibers [27] and starch-cellulose acetate wood flour composites [14]. These may result in higher strength properties as evident by the trends seen in strength properties of starch composites containing bagasse fibers. In fact, it is reported that aspect ratio of fibers also affects the overall properties of the biocomposites [4,16,17,23]. There are also reports stating that an increased aspect ratio results in higher tensile and flexural strength, as well as higher modulus in the case of composites of wood plastics [4,28]. Thus, a shorter fiber aspect ratio may result in lower strength properties as seen later in the next section. Polymorph granules of the matrix (Figure 6d) can be also seen on the fracture surface (Figure 6c) as well as on the fiber surface as in Figure 6b.

From all of the above fractographs (Figure 6 and Figure 8), an important observation is that the fracture surface of fibers of both banana and bagasse containing composites with both types of glycerin used (PA or RG) show more fiber ruptures parallel (longitudinal) to the surface. This is an interesting observation. It suggests that the interfaces still have some hold by the matrix over the fibers irrespective of the type of fiber. Also, the RG adheres better on the fiber surface than the PA glycerol (Figure 6c,d, Figure 7c and Figure 8f). This may be associated with greater availability of the plasticizing glycerin hydroxyl groups as well as from other polyols from the process of trans-esterification, which may establish more intense surface interactions. Figure 6e shows the non-structured starch details that presents small polymorph granules.

In general, the following things are to be noted: (i) the fibers in the composites would break at the fracture surface; (ii) due to good bonding between the matrix and the fiber, no broken fibers protruding from that surface would be detected. However, longer pull out fibers in the fracture surface of composites suggests weaker bonding [11] as seen in Figure 6b, Figure 7c and Figure 8f. Also, fiber failures occurring at the fiber–matrix interface is indicative of poor interfacial bonding, while its failure occurring within the matrix indicates good bonding [2,11]. Besides, good interfacial bonding is reflected by the non-existence of gaps between the fiber and the matrix [11]. In the case of biodegradable composites, particularly with starch based matrices, such bonding is considered chemically strong [12]. In the present case, also, this may be true, since COC contains some quantity of starch [43].

Further, it can also be seen that the COC + RG with bagasse fibers shows a different type of fracture with more wrinkles than that of composite containing banana fibers. This can probably be attributed to the dimensions of the bagasse fiber compared to that of the banana fibers. Also, the bagasse fibers were more uniformly distributed in comparison to that of banana. Such orientation is common when the pressure used to produce the composite is above 6 MPa [8]. Another feature of these composites is the dark brown colorations in the matrix of the fractured surface. This is probably be related to the type of starch present in the COC (ash tone in the case of dried, ripened and extracted) and the type of the glycerin used (liquid of intense dark yellow) due to the process of obtaining the “biodiesel” as reported elsewhere [14].

#### 3.7.2. Effect of Type of Glycerin Used

Fractured surfaces of COC cake with PA glycerol and 25% banana fiber are shown in Figure 7a–c. A smooth surface compared to that with RG (See Figure 8) can be seen along with the footprints of pullouts of fibers, which lie parallel to the fracture surface (shown by straight arrows).

This may induce fragility in this region. Pullout of fiber residue in COC can be seen as shown by the dotted arrow (a broken line with dots arrow indicates a fiber pullout). It is also observed that the composites containing crude glycerin were stiff and brittle like a ceramic material, while those with pure glycerol showed higher plasticity as evident from the status of the samples while testing.

At higher magnification (Figure 7b) of a region of Figure 7a, one can see brittle fracture of embedded fibers in the matrix without gaps existing between the constituents (matrix and fiber) of the composite tested indicating good bonding between them. The smoothness of the matrix surface is remarkable. Figure 7c shows the brittle nature of the fiber itself along with micro voids. Cracks in the matrix and a fiber pull out with micro fibrils forming channels indicate matrix coating on the fiber. However, no coating is observed on the fiber surface in this case.

Figure 8a is the fractured surface of the matrix mixture (70% COC with 30% RG samples) showing a slightly rough surface with very dark color. However, one can see homogeneous mixing of both constituents. Observed surface roughness here is understandable due to impurities present in the RG as well as due to small fibers present in COC.

These small fibers, which were found in the analyzed samples, are the ones retained during the simple crushing process of the mature castor bean oil seeds while getting the COC (starchy in nature). In fact, the observed structure and some small pores along with the presence of second phase in the structure justify this. Figure 8b shows matrix cracks (broken circle), micro voids (shown by small dotted arrows) and some fibers (shown by big dotted arrow). The micro voids seen here are probably due to the escape of gas due to evaporation of moisture during processing. Figure 8c is a higher magnification of Figure 8b. This shows internal region structure revealing hollow cavities with some globular material in some places.

On the other hand, with RG-25% banana fiber containing composites, one can see coating of the fiber surface in the fracture surface of the composite along with more fiber pull outs and more micro voids (Figure 8d–f). Footprints of fiber pullouts of fibers lying parallel to the fracture surface are evident in Figure 8d–f (shown by straight arrows).

Pulled out fibers perpendicular to the crack surface and a large number of pull out holes are also seen (shown by dotted and broken lines with arrows). Figure 7f shows a higher magnification image of the fractured surface revealing the coating of the whole surface of the pulled out fiber by the matrix along with brittle fractured fibers sitting pretty well within the matrix (shown by broken arrow). These fibers probably ruptured before the failure of the composite began to occur. The coating of banana fiber by COC RG matrix is in contrast to the coating of only the end of the fiber by COC + pure glycerin matrix, as well as in starch based composites observed earlier by the authors [27]. Hence, the observed lower trend of strength properties of banana fiber containing composites can be understood based on these observations.

## 4. Conclusions

- COC contained about 2.5% excess residual oil compared to the 1.5% reported in good quality solvent-extracted castor bean cakes.
- Moisture contents of COC, recovered glycerol (RG), and both banana and sugarcane bagasse fibers were found to be about 10.8%, 9.3%, 8.5%, and 9.2%, respectively.
- TGA/DTA results of the COC samples, both untreated and treated by solvent extraction, revealed them to be of different in nature in terms of mass loss and ash content.
- FTIR studies of recovered glycerol (RG) and pure glycerol revealed some differences between them in terms of appearance of vibrational bands (present only in RG) due to accumulation of residues from the alkaline trans-esterification process or coming from esters that were not properly separated from glycerin or due to long alkyl chain of fatty acids.
- The treatment of COC enhanced its crystallinity.
- Fiber incorporation significantly improved all the tensile properties of the composites, independent of fiber type.
- Fractographic studies of COC + glycerol (PA) or RG matrix with and without banana fibers revealed smooth fracture surface with the former and rough surface with the latter. In the case of composites, footprints of pullout of fibers lying parallel to the fracture surface have been observed suggesting inducement of fragility in this region.
- Compared to the stiff and brittle surface observed in COC + RG matrix containing fibers, that of COC + glycerol (PA) showed higher plasticity.
